# Naturopaths’ mobilisation of knowledge and information in clinical practice: an international cross-sectional survey

**DOI:** 10.1186/s12906-021-03383-2

**Published:** 2021-08-06

**Authors:** Amie Steel, Matthew Leach, Caragh Brosnan, Vicky Ward, Iva Lloyd

**Affiliations:** 1 Australian Research Centre in Complementary and Integrative Medicine, Faculty of Health, University of Technology Sydney, Ultimo, NSW Australia; 2 National Centre of Naturopathic Medicine, Southern Cross University, Lismore, NSW Australia; 3 School of Humanities and Social Science, College of Human and Social Futures, University of Newcastle, Callaghan, NSW Australia; 4 Reader in Management, School of Management, University of St Andrews, St Andrews, UK; 5 World Naturopathic Federation, Toronto, ON Canada

**Keywords:** Knowledge mobilisation, Knowledge translation, Evidence-based practice, Naturopathy

## Abstract

**Background:**

The contemporary evidence-based practice model acknowledges the importance of patient preferences and clinician experience when applying evidence within a clinical setting. Knowledge mobilisation (KM) acknowledges the complexities of knowledge translation by recognising and respecting diversity in types of knowledge and how such diversity can influence health care and health care choices. While there has been considerable discussion on KM in health care, it has received little attention in the field of naturopathy. Despite naturopathy’s widespread international use, it is unclear how naturopathic practitioners (NPs) use and share knowledge and information in clinical practice. This study examines the mobilisation of knowledge amongst NPs internationally.

**Methods:**

Online, international, cross-sectional survey of a self-selected sample of NPs from any country, that were either currently in clinical practice or had been in practice within the previous 12 months. The survey was administered in five languages (English, French, Portuguese, Spanish, German). Descriptive statistics were prepared for all survey items.

**Results:**

The survey was completed by 478 NPs who reported using an average of seven (median = 7, SD = 2.6) information sources to inform patient care. NPs also drew on knowledge gained through patients sharing their perspectives of living with their health condition (Always/Most of the time: 89.3%). They mostly sought knowledge about how a treatment might benefit a patient, as well as knowledge about treatment safety and a better understanding of a patient’s health condition. NPs frequently reported sharing knowledge developed through consideration of the patient’s unique needs (83.3%), and primarily shared knowledge by producing information for the public (72.6%) and for patients (72.2%).

**Conclusions:**

Based on these findings, it may be argued that NPs practice knowledge mobilisation; employing multiple forms and sources of knowledge, and mobilising knowledge to - as well as from - others. Due to their active engagement in patient and community education, NPs also may be considered knowledge brokers. In the context of the growing understanding of the complexities of knowledge translation and mobilisation in contemporary health care – and particularly within the context of implementation science – this study provides novel insights into an under-researched element of health services accessed by the community.

## Introduction

Evidence-based practice (EBP) is an important component of contemporary clinical decision-making and is integral to the provision of quality health care. The contemporary EBP model acknowledges the importance of patient preferences and clinician experience when applying evidence within a clinical setting [1]. Implicit within EBP is knowledge translation (KT). KT is a process whereby knowledge – primarily research evidence - is synthesised, exchanged and applied by relevant stakeholders [2] including, but not limited to, health practitioners. While there is some indication that KT may improve patient outcomes [3], opponents have argued for a broader view of knowledge and evidence in KT, which includes diversified perspectives and methods [4], and considers insights from grass-roots stakeholders [5]. A somewhat more collaborative model for KT - known as integrated knowledge translation (IKT) – responds to these concerns by promoting collaboration between researchers and knowledge users to identify the problem to be investigated. However, this model still places research as the preferred type of knowledge and researchers as critical to the knowledge production process [6].

Knowledge mobilisation (KM) acknowledges the complexities of KT by recognising and respecting diversity in types of knowledge and how such diversity can influence health care and health care choices [7]. Additionally, KM recognises the paternalism inherent in KT, whereby the researcher is viewed as generating all knowledge of value and the clinician is simply a recipient of that knowledge, which is then used to inform clinical decisions that patients blindly accept and follow without question [8]. In contrast, KM acknowledges different stakeholder values and emphasises the sharing of knowledge between parties to foster better health outcomes [7]. There has been considerable discussion on KM in health care, particularly in disciplines such as nursing, pharmacy, and physiotherapy [9, 10]. By contrast, KM has received little attention in the field of naturopathy. Naturopathy is recognised by the World Health Organisation as a traditional medicine system, is practiced in over 98 countries across all world regions, and is one of the more commonly used traditional health services in western countries [11]. Naturopathic practitioners (NPs) employ a range of therapies and treatments – most commonly including dietary changes, lifestyle prescription, nutritional products and herbal medicines - to manage their patients’ health concerns [12]. While such therapies and treatments are widely used by NPs, the practice of naturopathy is consistently defined worldwide by the application of six guiding principles that include explicit acknowledgement of the practitioner’s role in patient education and health promotion [13].

Despite the global presence of naturopathy, naturopathic medicine training and regulation vary considerably within and across countries [9], likely resulting in inconsistencies in clinical practices and patient outcomes. This is paralleled by diversity in information seeking behaviour, with naturopathic practitioners found to draw upon myriad information sources and knowledge - including published research [14–16], traditional knowledge sources [13, 16, 17], intuition [18], advice from peers [18], and clinical experience [18] - to inform their clinical decision-making [14, 15, 17]. Patients accessing naturopathic care also reportedly perceive NPs as using patient-provided knowledge to guide their treatment and to teach self-care to their patients [19, 20]. Even so, beyond the existing preliminary and localised research, it is unclear how information and knowledge is used and shared by NPs in their clinical practice. This study addresses this knowledge gap by examining the mobilisation of knowledge amongst NPs internationally. In doing so, the findings will help inform the development of pertinent strategies and/or interventions aimed at narrowing the gap between research and practice in naturopathic medicine, and in turn, help improve patient outcomes.

## Methods

### Design

Online, international, cross-sectional survey.

### Aim

The aim of the study was to identify the knowledge and information sources used and shared by NPs within the context of clinical practice.

### Participants and setting

The study included a self-selected sample of NPs from any country, that were either currently in clinical practice or had been in practice within the last 12 months. This included NPs on temporary leave from practice due to government restrictions from COVID-19 or personal leave (e.g., parental leave), as long as the period of leave did not exceed 12 consecutive months. Individuals were excluded if they were unable to complete the survey in any of the available languages.

#### Sample size

The study aimed to recruit a minimum of 385 study participants, which is in line with sample size calculations for descriptive survey research [21]. Participation rate was calculated based on the number of individuals who completed the first survey items pertaining to use of knowledge and information sources to inform clinical decision-making, divided by the number of participants who accessed the information sheet but did not respond to any survey items [22].

### Recruitment

A web-link to the online survey was shared via electronic direct mail to the NP membership of full association members of the World Naturopathic Federation (WNF), as well as social media platforms managed by WNF full members and the WNF office. Recruitment was undertaken between 12th September 2020 and 20th November 2020.

### Instrument

The survey was administered in five languages (English, French, Portuguese, Spanish and German) via Qualtrics™. The instrument included 122 core questions and an additional six adaptive questions, which were repeated up to nine times dependent on the number of items selected in one survey item (“Which of the following types of information sources do you employ when providing care to patients?”). The items were categorised into seven domains: 1 – *demographic and practice characteristics* (10 items); 2 - *practice behaviours* (21 items); 3 - *use of knowledge and information sources* (4 items); 4 - *use of, and attitudes towards, specific knowledge and information sources* (6 items repeated adaptively); 5 - *perceptions about knowledge and information sources* (36 items); 6 - *perceived stakeholder influence of knowledge use* (3 items); and 7 - *barriers to use of different knowledge types* (48 items). This analysis draws on participants’ responses to selected items from domains 1, 3 and 4.

#### Demographic and practice characteristics (domain 1)

Demographic items included participant age, gender, and country of practice. The survey also captured data on practice characteristics, including number of years since first naturopathic qualification, clinical practice environment (i.e., in clinical practice by self; in clinical practice with another health professional but no other naturopathic practitioners), average number of clinical practice hours per week, and average number of patient visits per week.

#### Use of knowledge and information sources (domain 3)

For the purposes of this survey, *knowledge* was defined as “a personal or individual familiarity, awareness or understanding, commonly gained through the experience of application or association”, while an *information source* was defined as “a resource from which knowledge or ideas are derived and may be shared and accessed by a wide range of people”. One survey item asked participants to identify the *information sources* used to inform the care they provided to their patients, with 11 response options presented (e.g. *information published in scientific journals by researchers, information provided by the patient*). A second survey item invited participants to select the *knowledge* they use to inform their care decisions, with eight response options presented (e.g., *knowledge developed during their initial clinical training, knowledge developed through clinical experience*). Additional survey items collected data pertaining to the methods participants use to share their own knowledge (e.g., *producing information to be published in scientific journal articles, producing information for patients*), and the types of knowledge they share with others (e.g., *knowledge developed through consideration of the patient’s unique needs, knowledge developed through clinical training*).

#### Use of, and attitudes towards, specific knowledge and information sources (domain 4)

Participants were exposed to a set of adaptive questions for each specific knowledge and information source selected from the items in domain three. These questions collected data on the frequency with which participants used each of the information sources selected (using a five-point Likert scale [Always – Never]), and the frequency they would prefer to use each of the information sources (using the same Likert scale). A further two items asked participants to indicate the type of knowledge (e.g., how a treatment works, the safety of a treatment) they sought from each selected information source, and how they use the knowledge they acquire from those sources (e.g., to share with a patient, to inform their own clinical decisions).

#### Instrument translation, validity and functionality

The instrument was tested for face validity and technical functionality by three individuals external to the research team and reflective of the target population. Minor amendments to item structure and survey flow were made based on feedback from the pilot testers. The invitation email, information sheet, and survey were all initially drafted in English and then translated into four other languages (i.e., French, Spanish, Portuguese, and German) using the automatic translation feature of Qualtrics™. The English and translated documents were then shared with native language speakers who cross-checked the translation for accuracy and meaning. Any further changes recommended by translators were re-checked by AS and IL using Google Translate. Where applicability of the changes was unclear, a second translator was invited to provide input until consensus was reached. All translations were coordinated by the World Naturopathic Federation.

### Data management and analysis

Data were exported from Qualtrics™ into Stata 16.1 (StataCorp LLC) for analysis. Items where participants were able to select all relevant response options were converted to ‘no’ responses for each response option where a participant response was missing, but a participant had selected one of the other options for that item. Missing data were excluded from the analysis. Data on country of location was categorized into world regions as defined by the World Health Organisation [23]. Descriptive statistics were prepared for all survey items (i.e., frequencies and percentages for categorical data, and means/medians and standard deviations/interquartile ranges for continuous data).

### Ethical clearance

This project was approved by the Human Research Ethics Committee of the University of Technology Sydney (#ETH20–5273).

## Results

### Participant characteristics

**Fig. 1:**
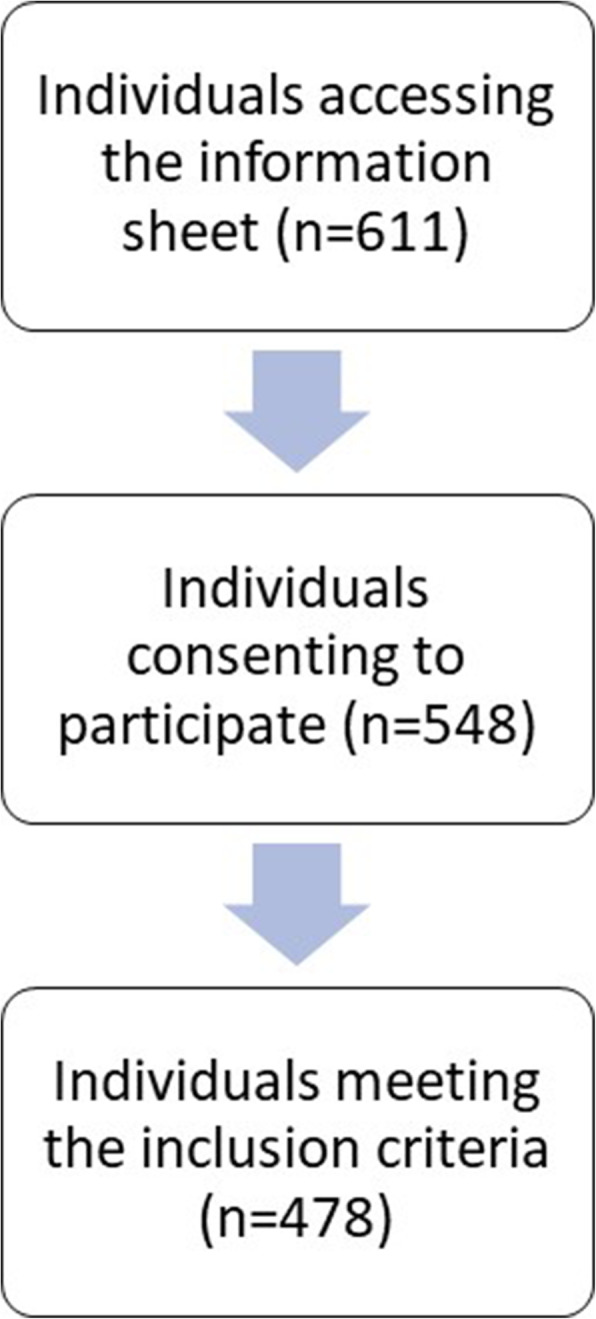
Flow chart demonstrating target population engagement and participant response rate

The survey achieved an 89.6% participation rate (*n* = 548) (see Fig. 1). Participants were on average 45.9 years old (SD 12.6) and most commonly identified as female-gendered (73.2%) (see Table 1). All world regions were represented with the greatest proportion of respondents located in North America (36.8%) and Western Pacific (23.2%). Approximately one-half (49.8%) of participants reported having been in practice between 5 and 10 years and more than one-third (37.2%) reported practicing in a clinic by themselves as their primary location of practice.

**Table 1:** Participant characteristics (*n* = 548)

	***Mean (SD)***	***Min, max***
Age (*n* = 520)	45.9 (12.6)	23, 81
	***n***	**%**
Gender (*n* = 530)		
*Male*	141	26.6
*Female*	388	73.2
*Non-binary*	1	0.2
World Health Region (*n* = 525)		
*North America*	193	36.8
*Latin America*	60	11.4
*Europe*	97	18.5
*Western Pacific*	122	23.2
*Africa/South East Asia/Eastern Mediterranean*	53	10.1
Years since first qualification (*n* = 486)		
*Less than 5 years*	120	24.7
*Between 5 and 10 years*	122	49.8
*Between 11 and 15 years*	78	16.1
*Between 16 and 20 years*	70	14.4
*More than 21 years*	96	19.8
Clinical practice environment		
*I am in a clinic by myself*	180	37.2
*I am in a clinic with other health professionals but no other naturopathic practitioners*	110	22.7
*I am in a clinic with other naturopathic practitioners but no other types of health professionals*	37	7.6
*I am in a clinic with other naturopathic practitioners and other health professionals*	119	24.6
*I am in a hospital setting*	10	2.1
*Other settings*	28	5.8
	***Mean (SD)***	***Min, max***
Clinical practice hours per week (*n* = 476)	22.6 (13.0)	1, 60
Patient visits per week (*n* = 481)	19.3 (17.7)	0, 130

### Naturopathic practitioners’ use of knowledge and information sources to inform patient care

The use of specific information sources to inform patient care is presented in Table 2. Participants most commonly reported using information published in scientific journals by researchers (76.2%) to inform the care provided to their patients, and most participants using this information source reported doing so ‘most of the time’ (44.4%). Information provided by the patient was selected as a source less often (64.6%), and the majority (81.7%) of participants using that information indicated they ‘always’ do so. Information from conferences and other professional events (74.1%) and information published in modern naturopathic clinical textbooks (70.7%) were also selected by the majority of participants, and were most commonly reported as being used ‘sometimes’ (conferences 30.8%, and modern naturopathic textbooks 34.5%). Overall, participants reported using an average of seven (median = 7, SD = 2.6) information sources to inform patient care (data not shown).

**Table 2:** Frequency of Information sources used by naturopathic practitioners to inform patient care, and frequency of source of knowledge and information shared by patients with naturopathic practitioners

	***n*** (%)	Frequency of use				
		Always	Most of the time	About half the time	Sometimes	Never
**Information source used by naturopaths to inform patient care (*n*** **= 478)**						
*Information published in scientific journals by researchers*	364 (76.2)	68 (19.2)	**157 (44.4)**	66 (18.6)	62 (17.5)	1 (0.3)
*Information gathered from conferences or other professional events*	354 (74.1)	31 (10.2)	87 (28.5)	**92 (30.2)**	**94 (30.8)**	1 (0.3)
*Information published in modern naturopathic clinical textbooks (published in the last 10 years)*	338 (70.7)	27 (9.0)	95 (31.8)	71 (23.8)	**103 (34.5)**	3 (1.0)
*Information from laboratory tests, pathology, or radiology tests*	335 (70.1)	78 (27.7)	**110 (39.0)**	50 (17.7)	44 (15.6)	0 (0.0)
*Information published in professional journals for clinicians*	333 (69.7)	31 (9.9)	**115 (36.9)**	81 (26.0)	83 (26.6)	2 (0.6)
*Information provided by the patient*	309 (64.6)	**205 (81.7)**	26 (10.4)	9 (3.6)	11 (4.4)	0 (0.0)
*Information published in general clinical textbooks*	296 (61.9)	24 (8.9)	87 (32.1)	59 (21.8)	**100 (36.9)**	1 (0.4)
*Information from clinical guidelines*	248 (51.9)	24 (12.2)	**85 (43.2)**	31 (15.7)	54 (27.4)	3 (1.5)
*Information provided by product companies*	230 (48.1)	7 (3.5)	43 (21.5)	51 (25.5)	**99 (49.5)**	0 (0.0)
*Information published in traditional naturopathic clinical textbooks (published more than 50 years ago)*	193 (40.4)	6 (3.5)	46 (27.1)	24 (14.1)	**87 (51.2)**	7 (4.1)
**Source of knowledge or information shared by patients with their naturopathic practitioner**						
*Patients perspective of living with their condition (n = 371)*		**182 (49.1)**	149 (40.2)	27 (7.3)	13 (3.5)	0 (0.0)
*Patient’s personal health history (n = 371)*		**166 (44.9)**	126 (34.1)	47 (12.7)	29 (7.8)	2 (0.5)
*Patient’s family health history (n = 371)*		101 (27.2)	**152 (41.0)**	54 (14.5)	62 (16.7)	2 (0.5)
*Conventional medical examinations or tests (n = 371)*		77 (20.8)	**164 (44.2)**	75 (20.2)	51 (13.8)	7 (1.1)
*Functional examinations or tests (*e.g.*, urine/salivary hormone tests) (n = 369)*		48 (13.0)	77 (20.9)	78 (21.1)	**154 (41.7)**	12 (3.3)
*General internet sources (*e.g.*, blogs, social media) (n = 370)*		36 (9.7)	**154 (41.6)**	93 (25.2)	79 (21.4)	8 (2.2)
*Other health professionals involved in their care (n = 371)*		21 (5.7)	107 (28.8)	115 (31.0)	**125 (33.7)**	3 (0.8)
*Informal sources (*e.g.*, family and friends) (n = 371)*		20 (5.4)	83 (22.4)	113 (30.5)	**144 (38.8)**	11 (3.0)
*Books (n = 371)*		9 (2.4)	46 (12.4)	71 (19.2)	**229 (61.7)**	16 (4.3)
*Broadcast media (*e.g.*, TV, radio) (n = 370)*		12 (3.2)	63 (17.0)	64 (17.3)	**196 (53.0)**	35 (9.5)
*Research organisations (n = 368)*		5 (1.4)	10 (2.7)	8 (2.2)	**208 (56.5)**	137 (37.2)
*Patient advocacy or support groups (n = 371)*		4 (1.1)	13 (3.5)	32 (8.6)	**219 (59.0)**	103 (27.8)
*Government agencies (n = 369)*		1 (0.3)	9 (2.4)	23 (6.2)	**217 (58.8)**	119 (32.3)
*Published journal articles (n = 371)*		1 (0.3)	19 (15.1)	13 (3.5)	**217 (58.5)**	121 (32.6)

The knowledge types participants reportedly used to inform patient care included knowledge developed through clinical experience (86.2%, *n* = 412) or initial clinical training (81.2%, *n* = 388), continuing professional education delivered by an expert clinician (79.9%, *n* = 382), consideration of the patient’s unique needs (78.7%, *n* = 376), and discussions with professional peers (75.7%, *n* = 362). Less common knowledge types used by participants were knowledge developed through continuing professional education delivered by a researcher (59.8%, *n* = 286) and discussions with a mentor or expert (55.4%, *n* = 265).

### Source of knowledge and information shared by patients with naturopathic practitioners

Table 2 presents respondent perceptions regarding the frequency with which the patient shares knowledge or information drawn from specific sources with the NP. The patient’s perspectives of living with their health condition (Always: 49.1%, Most of the time: 40.2%) and the patient’s personal health history (Always: 44.9%; Most of the time: 34.1%) were identified as the most frequently used knowledge or information sources shared by the patient. The patient’s family health history (Always: 27.2%; Most of the time: 41.0%) and conventional medical examinations or tests (Always: 20.8%; Most of the time: 44.2%) were also commonly reported, although not as frequently. Knowledge and information from general internet sources were also reported as shared by patients ‘most of the time’ (41.6%). Other sources – including other health professionals involved in their care, broadcast media, research organisations and government agencies - were most reported as ‘sometimes’ mentioned by patients. The sources most frequently reported as ‘never’ mentioned by patients were research organisations (37.2%), published journal articles (32.6%), government agencies (32.3%) and patient advocacy or support groups (27.8%).

### Type and application of knowledge sought from information sources

Table 3 presents the knowledge that participants sought from each information source they used. Knowledge about how a treatment might benefit a patient was the most common type of knowledge that participants sought from information gathered from conferences or other professional events (92.5%), product companies (88.0%), professional journals for clinicians (91.0%), scientific journals by researchers (87.9%), traditional naturopathic clinical textbooks (90.5%) or modern naturopathic clinical textbooks (87.5%). Clinical guidelines were predominantly used to seek knowledge of the safety of a treatment for a patient (82.7%), but this knowledge was more commonly sought from scientific journals. Information from laboratory, pathology, or radiology tests (92.6%), information provided by the patient (94.8%), and information published in general clinical textbooks (84.5%) were used by most participants to seek knowledge to understand the patient’s health condition. The most common reason for using the different information sources was informing the participant’s own clinical decision for an individual patient. This was the least common reason for using information published in general clinical textbooks, with a similar proportion of participants (67.3%) reporting the use of that information source to share knowledge with their patient.

**Table 3:** Knowledge sought from each information source, and how the knowledge is applied [presents results from respondents that reported using this information source]

Information source used	Knowledge sought from the information source					Application of knowledge from the information source					
	How a treatment works	How a treatment might benefit my patient	The safety of a treatment for my patient	Understanding of my patient’s health condition	Understanding about treatments of my patient has been prescribed by another health professional	To share with my patient	To share with other health professionals	To inform my own clinical decisions for an individual patient	To share with the general community	To develop clinical practice guidelines or policies for a group of patients	To produce useful research or scientific knowledge
Information published in scientific journals by researchers (*n* = 354)	279 (78.8)	**311 (87.9)**	296 (83.6)	242 (68.4)	234 (66.1)	283 (80.2)	187 (53.0)	**322 (91.2)**	136 (38.5)	117 (33.1)	106 (30.0)
Information gathered from conferences or other professional events (*n* = 305)	250 (82.0)	**282 (92.5)**	236 (77.4)	229 (75.1)	184 (60.3)	216 (71.0)	151 (49.7)	**279 (91.8)**	108 (35.5)	111 (36.5)	49 (16.1)
Information published in modern naturopathic clinical textbooks (published in the last 10 years) (*n* = 297)	226 (76.1)	**260 (87.5)**	229 (77.1)	204 (68.7)	135 (45.5)	213 (71.5)	117 (39.3)	**262 (87.9)**	96 (32.2)	93 (31.2)	54 (18.1)
Information from laboratory tests, pathology, or radiology tests (*n* = 282)	65 (23.0)	133 (47.2)	123 (43.6)	**261 (92.6)**	106 (37.6)	228 (81.1)	85 (30.3)	**264 (94.0)**	21 (7.5)	47 (16.7)	40 (14.2)
Information published in professional journals for clinicians (*n* = 312)	265 (84.9)	**284 (91.0)**	262 (84.0)	226 (72.4)	199 (63.8)	240 (76.9)	152 (48.7)	**290 (93.0)**	114 (36.5)	100 (32.1)	78 (25.0)
Information provided by the patient (*n* = 252	65 (25.8)	130 (51.6)	114 (45.2)	**239 (94.8)**	158 (62.7)	106 (42.4)	53 (21.2)	**234 (93.6)**	19 (7.6)	40 (16.0)	33 (13.2)
Information published in general clinical textbooks (*n* = 271)	193 (71.2)	205 (75.7)	186 (68.6)	**229 (84.5)**	152 (56.1)	**182 (67.3)**	97 (15.9)	**210 (67.3)**	88 (32.6)	75 (27.8)	56 (20.7)
Information from clinical guidelines (*n* = 196)	107 (54.6)	145 (74.0)	**162 (82.7)**	105 (53.6)	109 (55.6)	126 (64.6)	81 (41.5)	**174 (89.2)**	54 (27.7)	83 (42.6)	38 (19.5)
Information provided by product companies (*n* = 200)	146 (73.0)	**176 (88.0)**	166 (83.0)	62 (31.0)	70 (35.0)	132 (66.0)	59 (29.5)	**175 (87.5)**	41 (20.5)	47 (23.5)	22 (11.0)
Information published in traditional naturopathic clinical textbooks (published more than 50 years ago) (*n* = 169)	117 (69.2)	**153 (90.5)**	86 (50.9)	89 (52.7)	59 (34.9)	115 (68.5)	71 (42.3)	**142 (84.5)**	55 (32.7)	33 (19.6)	26 (15.5)

### Methods of knowledge-sharing and types of knowledge shared by naturopathic practitioners

The most common type of knowledge that participants reported sharing with others was knowledge developed through consideration of the patient’s unique needs (83.3%) and knowledge developed during their clinical training (81.1%) (see Table 4). The least common knowledge type shared with others was knowledge developed through continuing professional education delivered by a researcher (61.9%). Participants reported using a range of methods to share their knowledge, including producing information for the general public (72.6%) and for patients (72.2%). Sharing knowledge by producing information for publication in scientific (18.3%) or naturopathic (18.0%) journal articles was less common as was sharing knowledge by producing information for publication in naturopathic (11.4%) or general (8.9%) clinical textbooks.

**Table 4:** Methods of knowledge-sharing and types of knowledge shared by naturopathic practitioners

	***n***	%
**Knowledge types shared (*n*** **= 449)**		
*Knowledge developed through consideration of the patient’s unique needs*	374	83.3
*Knowledge developed during your initial clinical training*	364	81.1
*Knowledge developed through continuing professional education developed by an expert clinician*	348	77.5
*Knowledge developed through discussions with professional peers*	336	74.8
*Knowledge developed through continuing professional education delivered by a researcher*	278	61.9
**Methods of knowledge sharing (*n*** **= 438)**		
*Producing information for the general public (*e.g. *social media, blogs, community talks, magazine talks)*	318	72.6
*Producing information for patients (*e.g. *information handouts, newsletters)*	316	72.2
*Producing information delivered through clinical training for naturopathic students*	142	32.4
*Producing information delivered through continuing professional education events for other clinicians*	123	28.1
*Producing information to be published in scientific journal articles*	80	18.3
*Producing information to be published in naturopathic journal articles*	79	18.0
*Producing information to be published in modern naturopathic clinical textbooks*	50	11.4
*Producing information to be published in general clinical textbooks*	39	8.9
*Producing information for product companies*	39	8.9

## Discussion

This study presents an international examination of the knowledge and information sources used and shared by NPs within the context of clinical practice, and in doing so uncovers a number of important findings, as discussed below.

### NPs draw knowledge from varied information sources

The results highlight the variety and complexity of knowledge NPs use and share to inform their clinical practice. Previous qualitative research suggests that while NPs use evidence-based procedures in the same way as other professions, they may be less likely to refer to the concept of EBP [24]. Our study delves behind this broad label to ascertain the information sources NPs use to derive knowledge for their clinical practice, and how they use them. The finding that NPs use an average of seven information sources to inform patient care means that the EBP framework - in which published information (primarily research), clinical experience and patient preference are triangulated [1] – accounts for only a portion of the knowledge translation process taking place. Instead, NPs are drawing on and influenced by diverse information sources, including product companies.

Among the information sources used to inform care, information published in scientific journals was the most widely used by NPs. This finding departs from earlier research reporting that complementary medicine practitioners prefer traditional knowledge and textbooks [14, 25]. The difference may reflect a change over time and higher uptake of EBP, geographical differences, or that NPs are more likely to apply evidence from journals than other complementary medicine practitioners studied. However, further research is needed to understand how NPs engage with journal publications and apply the information, given previous findings that many practitioners have limited access to full-text journals [15]. It is worth noting that nearly one-quarter of respondents do not report using information from scientific journals to inform patient care, suggesting that the uptake of research findings may be still limited compared with what has been observed for other health professions [25, 26]. Previous research suggests the barriers to NPs using published research to inform their clinical practice include poor transferability of new knowledge from research due to misalignment between the design of interventions and routine daily naturopathic practice [16], poor access to full-text articles, or limited research appraisal skills [14, 15].

Conferences and professional events were used as information sources almost as commonly as scientific journals. The nature of these events may not completely align with the definition of information source as they are, in part, characterised by social interactions between attendees that provide an opportunity for knowledge generation. Previous Australian research by Braun et al. revealed 67% of NPs used conferences and seminars as an information source for commonly used complementary medicines [27]. However, other qualitative research suggests that information derived from these sources may be viewed with some wariness by NPs, particularly if they are provided by product manufacturers [15]. Indeed, our study and previous research by Braun et al. found information provided by product companies was among the least frequently used by NPs [27], suggesting the findings from the previously mentioned qualitative study may also apply here.

Modern clinical textbooks were another important resource for respondents. Previous research has found NPs use modern clinical textbooks to locate specific information such as drug interactions and pathophysiology of health conditions [15]. Traditional textbooks were used less frequently but still used by a significant minority to inform clinical decisions and determine how a treatment might benefit a patient. It is also interesting to note that NPs frequently use clinical practice guidelines (CPGs) to inform care, even though CPGs generally provide limited guidance on complementary medicine interventions [28]. This finding is likely to be explained through further qualitative investigation.

### Importance of the patient experience

Patients are a source of information for more than two-thirds of participants and an information source reportedly used ‘always’ by the highest proportion of users. Prior qualitative research reports that NPs see comprehensive case history-taking as crucial in understanding the patients’ experience of symptoms [24], and our study supports this finding, revealing that patients’ personal health histories are shared with the vast majority of practitioners, 'always' or 'most of the time'. Furthermore, previous surveys have found 97% of NPs considered patient reports or feedback as essential or important to clinical practice [27].

Over and above the patient’s history is the patient’s perspective of living with the condition, which was the form of knowledge patients most often shared with practitioners in our study. The role of this less structured, more experiential knowledge has largely been excluded from formulations of EBP, where the patient perspective is typically reduced to the patient’s preference among a set of discreet choices presented by the clinician [29]. While the clinician’s experience is explicitly included in EBP, the patient’s experience is not [1]. Indeed, in the evidence hierarchy, patients’ individual experiences are framed as anecdotal, positioning them at the bottom [29]. In contrast, Greenhalgh et al. argue that ‘the richness of narrative’ – that is, listening to the patient’s story – is essential information required to appropriately tailor research-based treatment to an individual case [29].

Although our data do not determine how the patient perspective (versus the patient history) is deployed by NPs, the prominence of this form of knowledge exchange suggests that patients are typically given space in the consultation to share their stories. This finding adds to previous research showing that patients largely feel seen and heard as unique individuals by NPs and that NPs are geared towards patient-centred care [19]. All in all, this finding points to the value placed by NPs on the patient experience and the cultivation of a therapeutic relationship that elicits such knowledge-sharing from patients [30]. Again, while the quality of the therapeutic relationship is not generally factored into EBP frameworks, it is a crucial factor in the capacity to successfully mobilise research-based evidence in a clinical encounter [29] and to respond to the growing imperative in global policy to build patient-centred care into health systems [31]. Notwithstanding, further research is needed to better understand NPs’ perspectives on the role of patient experiences in knowledge exchange.

Our study shows that knowledge from most information sources was used to inform NP treatment decisions, while patient information was used primarily to provide the clinicians with a better understanding of the patient’s health condition. This may signal a distinction being made by practitioners around the validity of different knowledge types, with earlier qualitative research showing NPs do not always view patient-provided information as reliable [15].

### The sharing of knowledge with patients and the public

Another significant form of KM for NPs was the sharing of knowledge with patients and the general public, with most respondents doing so. Sharing knowledge with patients aligns with the naturopathic principles of patient education and health promotion [13], and our results suggest general adoption of these principles across the international profession. Sharing knowledge with the general public, such as through social media, was the most common method of knowledge-sharing. Indeed, the results demonstrated that NPs very often assume a KT role that reaches beyond their local patient base, perhaps more so than other professions. With this in mind, it may be argued that NPs play a role as knowledge broker – an individual that promotes interaction between researchers and end users [32] - to a sector of the community that may be underserved by mainstream health services. However, as is the case in many areas of implementation science, the current focus of knowledge brokerage is on the broker’s role in translating evidence to policy and practice [33]. In the case of NPs, the primary emphasis appears to be translating evidence for sharing with patients and the public.

It has been argued that engaging with online patient communities is an important part of contemporary knowledge exchange that has been overlooked in EBP [29], and further investigation of NPs’ activity in this space may highlight strategies that other professions could learn from. One qualitative study specifically examining NPs’ blog-writing found blogs were used for a range of purposes, including helping raise the profession’s profile and sharing its core ideals, as part of efforts to establish credibility and contribute to ‘a growing public narrative about their discipline’ [34]. Such uses may explain why, apart from knowledge of patients’ unique needs, information from initial clinical training was the type most often shared by NPs in our study - perhaps as a way of carving out a distinctive professional niche in what is now a crowded online space of health information-sharing. Additional research is needed to examine other ways NPs engage the general public in knowledge exchange.

### Limitations

One limitation of this study is that it does not capture how NPs are combining knowledge types or information sources. Previous research has concluded that NPs integrate knowledge types through a form of deductive reasoning in practice, whereby gaps in one source of information are addressed through another [18]. More recently, it has been argued that health professionals actually create and draw on hybridised knowledge, arrived at via a complex process in which multiple internal and external forms of knowledge are brought together dialogically [35]. It is not possible to tell from our data whether and when different forms of knowledge are being applied discreetly by NPs or, as is more likely, combined in one of these ways. Further work is needed to develop a way of quantitatively studying these knowledge integration practices uncovered in qualitative studies. The study is also limited by the respondent sample, with the majority of participants having been in practice for less than 10 years. This could skew the results as graduates of more recent naturopathic educational programs are more likely to be exposed to EBP in the curriculum [17]. Additionally, the vast majority of study participants come from North and South America, Europe and the Western Pacific, and findings cannot easily be generalised to other world regions with small numbers of respondents.

## Conclusion

This study found NPs draw knowledge from a diverse range of information sources. While published research evidence is prominent among them, they also draw on traditional knowledge, clinical experience and patient expertise regarding their own health condition. NPs also appear to be active in sharing their knowledge with patients and the wider community. Based on these findings, it may be argued that NPs practice knowledge mobilisation; employing multiple forms and sources of knowledge, and mobilising knowledge to - as well as from - others. Due to their active engagement in patient and community education, NPs also may be considered knowledge brokers. In the context of the growing understanding of the complexities of knowledge translation and mobilisation in contemporary health care – and particularly within the context of implementation science – this study provides novel insights into an under-researched element of health services accessed by the community.

## Data Availability

The datasets used and/or analysed during the current study are available from the corresponding author on reasonable request.

## Abbreviations

KM: Knowledge mobilisation
NPs: Naturopathic practitioners
EBP: Evidence-based practice
KT: Knowledge translation
IKT: Integrated knowledge translation
WNF: World Naturopathic Federation
CPGs: Clinical practice guidelines
